# Using NEURON for Reaction-Diffusion Modeling of Extracellular Dynamics

**DOI:** 10.3389/fninf.2018.00041

**Published:** 2018-07-10

**Authors:** Adam J. H. Newton, Robert A. McDougal, Michael L. Hines, William W. Lytton

**Affiliations:** ^1^Department of Neuroscience, Yale University, New Haven, CT, United States; ^2^SUNY Downstate Medical Center, The State University of New York, New York, NY, United States; ^3^Center for Medical Informatics, Yale University, New Haven, CT, United States; ^4^Neurology, Kings County Hospital Center, Brooklyn, NY, United States

**Keywords:** reusability, computer simulation, multiscale modeling, spreading depression, stroke

## Abstract

Development of credible clinically-relevant brain simulations has been slowed due to a focus on electrophysiology in computational neuroscience, neglecting the multiscale whole-tissue modeling approach used for simulation in most other organ systems. We have now begun to extend the NEURON simulation platform in this direction by adding extracellular modeling. The extracellular medium of neural tissue is an active medium of neuromodulators, ions, inflammatory cells, oxygen, NO and other gases, with additional physiological, pharmacological and pathological agents. These extracellular agents influence, and are influenced by, cellular electrophysiology, and cellular chemophysiology—the complex internal cellular milieu of second-messenger signaling and cascades. NEURON's extracellular reaction-diffusion is supported by an intuitive Python-based *where/who/what* command sequence, derived from that used for intracellular reaction diffusion, to support coarse-grained macroscopic extracellular models. This simulation specification separates the expression of the conceptual model and parameters from the underlying numerical methods. In the volume-averaging approach used, the macroscopic model of tissue is characterized by *free volume fraction*—the proportion of space in which species are able to diffuse, and *tortuosity—*the average increase in path length due to obstacles. These tissue characteristics can be defined within particular spatial regions, enabling the modeler to account for regional differences, due either to intrinsic organization, particularly gray vs. white matter, or to pathology such as edema. We illustrate simulation development using spreading depression, a pathological phenomenon thought to play roles in migraine, epilepsy and stroke. Simulation results were verified against analytic results and against the extracellular portion of the simulation run under FiPy. The creation of this NEURON interface provides a pathway for interoperability that can be used to automatically export this class of models into complex intracellular/extracellular simulations and future cross-simulator standardization.

## 1. Introduction

Computational neuroscience has had an historical focus on electrophysiology, with consequent neglect not only of the accompanying chemophysiology that directly underlies neural function, but also of the brain as a complex organ within which neuronal networks are embedded (De Schutter, 2008). This neglect is of particular importance as we try to adapt our models for understanding of brain disease, many of which are associated with changes in extracellular concentrations of ions, metabolites, transmitters, or toxins in various parts of the brain (Mulugeta et al., 2018). These extracellular concentration changes then cause alterations in reactions and reaction rates involving cellular elements including specific and nonspecific receptors, ion channels, and intracellular signaling pathways. In order to begin to fill out modeling of the brain as a whole organ, we have developed an extracellular modeling extension for the NEURON modeling platform (Carnevale and Hines, 2006), a widely used simulation tool that has been used in over 1900 neuroscience publications, with around 600 models freely available on ModelDB (McDougal et al., 2017).

NEURON has always allowed modelers to describe arbitrarily complex phenomena with their own “mod” files, optionally including verbatim C-code, thereby permitting arbitrary programming to be done to augment the package. This left the user with complex code which intermingled model specifics with the numerics, making reuse difficult. One of the guiding principles of simulator development, both for NEURON and for other simulators, has been to promote reproducibility, reusability, and credibility by providing a consistent numerics-independent way to specify models. In the reaction-diffusion domain, the NEURON *rxd* module simplified and standardized the description of accumulation, reaction and diffusion (McDougal et al., 2013). This module has been used to study calcium dynamics in both physiological and pathological conditions (Neymotin et al., 2014, 2016). We have now expanded the *rxd* module to include macroscopic volume averaged description of extracellular space (ECS). This is appropriate for spatial discretization on the order of 10 μm to produce simulations up to centimeters (Nicholson and Phillips, 1981; Nicholson, 1995). The *rxd* macroscopic model of tissue is parameterized by free volume fraction—the proportion of space unoccupied by cells, blood vessels, etc.; and tortuosity—the increase in a diffusing particle's path-length due to obstacles.

In the following sections we give details of the development of the extracellular *rxd* module, with examples to demonstrate the utility of the Python interface. We then show some details of the numerical methods underling the module's interface and techniques used to improve performance for large simulations, providing several tests to verify *rxd* simulation results. We give a basic example of clinically-relevant simulation by demonstrating the phenomenon of *spreading depression*, a pathological condition thought to play a role in a variety of conditions including mirgraine, epilepsy, and stroke (Wei et al., 2014).

## 2. Objectives

As with cells of other solid organs, neurons exist in a highly active medium, influenced by bioactive chemicals whose concentrations change rapidly through: (1) passive diffusion, (2) active deposit and clearance from other cells, and (3) binding or other reactions with extracellular species (Syková and Nicholson, 2008). These important tissue-level *chemophysiological* influences have been neglected by computational neuroscience for a variety of reasons, including the aforementioned focus on electrophysiology. Primarily, however, simulators have been unable to support this level of interaction due to the difficulty of reconciling the small spatial scale of single cell and local network simulation with the large millimeter (mouse) or centimeter (primate) scale of the brain as an organ. This type of broad multiscale modeling naturally requires compromises at both ends, and across the temporal scales as well. We set out to extend NEURON to handle this domain by providing a coarsely-discretized extracellular domain within which cells and networks can be embedded, creating *mosaic models* where different parts are provided at different levels of detail. The coarse scale permits relatively rapid simulation runs, but is sufficiently detailed to set parameters based on currently available experimental measures. Other spatial scales will be added to this mosaic in the future.

A major focus for both the original *rxd* module and this extension is ease-of-use. This goal is partly achieved by separating the user from the details of the numerics enhancing reproducibility by making it easy to identify the conceptual model. Additionally, the *rxd* Python interface subserves this goal by providing relatively simple, biologically-intuitive representations that allow the user to focus on the translation of the conceptual model by specifying (1) regions: *where?* — in this case the ECS; (2) species in each region: *who?* — an ionic species, a peptide, a transmitter, etc.; and (3) transformations *what?* — reactions between species, signaling across a membrane, or transits involving the same species across a membrane.

Providing consistent modeling of both intracellular and extracellular space also ensures conservation of mass. The total amount of a substance of interest will be conserved within the simulation, despite moving in and out of subcellular compartments, or in and out of cells, via currents, active transport, or vesicular release.

## 3. Examples

We present two related examples to demonstrate the use of the *rxd* module to model extracellular concentrations: (1) simple potassium diffusion, and (2) spreading depression. In each case we begin by specifying the region for the dynamics, here the ECS. We then identify the species involved. Finally, their interactions with each other or with fixed agents are identified. The code for these examples are available at ModelDB (http://modeldb.yale.edu/238892) (McDougal et al., 2017).

### 3.1. Potassium diffusion in ECS

This example shows potassium diffusion through a box of ECS, with spatial uptake represented phenomenologically as a reaction. We demonstrate each of the stages required to specifying a model. First, to use extracellular *rxd*, we import it from NEURON and enable it:


from neuron import crxd as rxd
rxd.options.enable.extracellular = True


#### 3.1.1. Region

We then specify the specific extracellular region;


ecs = rxd.Extracellular(xlo=-500, ylo=-500,
    zlo=-500, xhi=500, yhi=500,
    zhi=500, dx=10,
    volume_fraction=0.2,
    tortuosity=1.6)
 

(xlo,ylo,zlo) and (xhi,yhi,zhi) define the lower left back and upper right front corners of a 3D box in micrometers. dx is the size of a side of a cubic voxel; alternatively dx can be a 3-element tuple to specify voxel length, height and depth. The optional argument volume_fraction is the free volume fraction or porosity, the accessible portion of extracellular volume. The tortuosity is the average multiplicative increase in path length a particle must travel due to obstacles. The effective diffusion coefficient is the free diffusion coefficient divided by the square of the tortuosity. Here, the free volume fraction (0.2) and tortuosity (1.6) were set to typical values for brain (Syková and Nicholson, 2008). Both the volume fraction and the tortuosity can be scalar values as shown here. Alternatively, arrays the size of the extracellular space, or functions that take the *x*, *y*, *z* coordinates as arguments can be used (section 3.2.1). Extracellular concentrations are given relative to free volume, i.e., the total amount in a voxel divided by free volume of the voxel.

#### 3.1.2. Species

To create extracellular potassium, we use the same rxd.Species call as would be used for intracellular diffusion; the difference is in the first argument that gives the extracellular region.


k = rxd.Species(ecs, name='k',
    d=2.62, charge=1,
    initial=lambda nd: 40
      if nd.x3d**2 + nd.y3d**2 + nd.z3d**2
      < 100**2 else 3.5,
    ecs_boundary_conditions=3.5)


Where d (the free diffusion coefficient) is set to 2.62μm^2^/ms for K^+^ (Samson et al., 2003), where *d* has been increased to reflect a higher temperature of 37°C by using the Stokes-Einstein equation, assuming viscosity of the extracellular fluid to be the same as water. Anisotropic diffusion is supported by passing a 3-tuple for diffusion coefficients in 3 dimensions. Initial conditions can be a scalar value for the whole region, an array matching the region $\left(\text{i}.\text{e}.,\;\left\lceil \frac{xhi-xlo}{dx}\right\rceil ,\;\left\lceil \frac{yhi-ylo}{dy}\right\rceil ,\;\left\lceil \frac{zhi-zlo}{dz}\right\rceil \right)$ or an anonymous (lambda) function, as shown here. The lambda function is given a NodeExtracellular as argument, allowing the model to specify initial concentration depending on the location (x3d, y3d, z3d). If the species exists in both intracellular rxd.Region and the ECS then the initial function will receive both NodeExtracellular and either Node1D or Node3D from the class rxd.node. This multiplicity of regions, where the same location is represented in both the intracellular space and the ECS is due to using an interposition of intracellular and extracellular space handled by ECS free volume fraction, instead of by using excluded volume. The initial function can assign values by first checking region is equal to the defined ecs. The default boundary conditions for the ECS are Neumann (zero flux). Dirichlet boundary conditions can be specified with the keyword argument ecs_boundary_conditions set to the desired concentration. Concentrations are in mM.

#### 3.1.3. Reactions

Extracellular reactions are specified using rxd.Rate, rxd.Reaction and rxd.MultiCompartmentReaction as described in the *rxd* tutorial (McDougal, 2018). We consider the case of excess potassium in the ECS, which is primarily taken up by astrocytes (MacAulay and Zeuthen, 2012). A wide variety of modeling options are available for explicitly modeling these cells at various levels of complexity (Wei et al., 2014; Conte et al., 2018). Here we demonstrate the phenomenological model of astrocytic buffering from (Bazhenov et al., 2004; Krishnan and Bazhenov, 2011). This model treats astrocytes as a chemical buffer that could take up excess K^+^ but would then release K^+^ when ECS levels dropped.

(1) $$\begin{array}{l} \left[K][A]\overset{k_{f}}{\underset{k_{b}}{⇄}}[AK\right] \end{array}$$

where *A* is the concentration of free astrocyte “buffering” capacity and *AK* is the concentration of bound potassium. By default mass-action kinetics are assumed, so the stoichiometry is implicit. The rate of change in unbound astrocyte capacity A used in the following example is then given by; kf*[K]*[A] - kb[AK] mM/ms. Alternative kinetics can be specified with the keyword argument mass_action=False. The rates would then be assumed to be the full forward and reverse rates, and change in unbound buffer would be kf-kb mM/ms. The initial condition Amax represents the total capacity of glial to buffer K^+^ (in mM), in this phenological model it represents the density of astrocyte uptake/binding sites. These sites are immobile: d=0.

The specification of kf uses the exponential of an rxd.Species. This is achieved in Python by importing the rxd.rxdmath module, which provides the same library of functions as the Python math module. However while Python math functions require numeric arguments the rxd.rxdmath allows rxd.Species to be used, as in the following example;


from neuron.rxd import rxdmath
kb = 0.0008   #backward rate mM/ms
kth = 15.0    #k threshold
kf = kb/(1.0 + rxdmath.exp(-(k - kth)/1.15))
Amax = 10 #uptake/release site density
  
A = rxd.Species(ecs,name='astrocyte',
    d=0, initial=Amax)
AK = rxd.Species(ecs,name='bound',
    d=0, initial=0)
  
astrocytes = rxd.Reaction(k + A, AK, kf,
              kb)


### 3.2. Cortical spreading depression

The preceding simulation framework can be used to develop a model of spreading depression (SD). SD is a wave of near complete depolarizations of neurons that propagates in gray matter at 2–7 mm/min and lasts for ~1 min. This phenomena is highly reproducible and is associated with several pathological conditions, including; migraines, ischemic stroke, traumatic brain injury and epilepsy (Somjen, 2004). An early mechanistic model attributed the depolarization to an increase in extracellular K^+^ (Grafstein, 1956). A positive feedback loop underlies SD: high extracellular K^+^ activates cells whose depolarization opens K^+^ channels which release more K^+^ into extracellular space.

To produce this positive feedback between ECS and cellular physiology, we simulate a realistic density of 90,000 cells/mm^3^ embedded in 1mm^3^ of ECS with diffusion of both K^+^ and Na^+^. Each neuron has a soma and dendrite with the Hodgkin-Huxley complement of channels (naf, kdr, gleak) as well as kleak and nap (persistent Na^+^ channel) with parameters based on Conte et al. (2018). This initial simplified model omits several mechanisms likely to contribute to spreading depolarization, including slow Ca^2+^-dependent K^+^ currents. More importantly, we omit neurons Na-K-ATPase, a major mechanism for restoring ion gradients. As noted above, glial Na-K-ATPase is partially modeled by the field of K^+^ sink.

**Figure 1:**
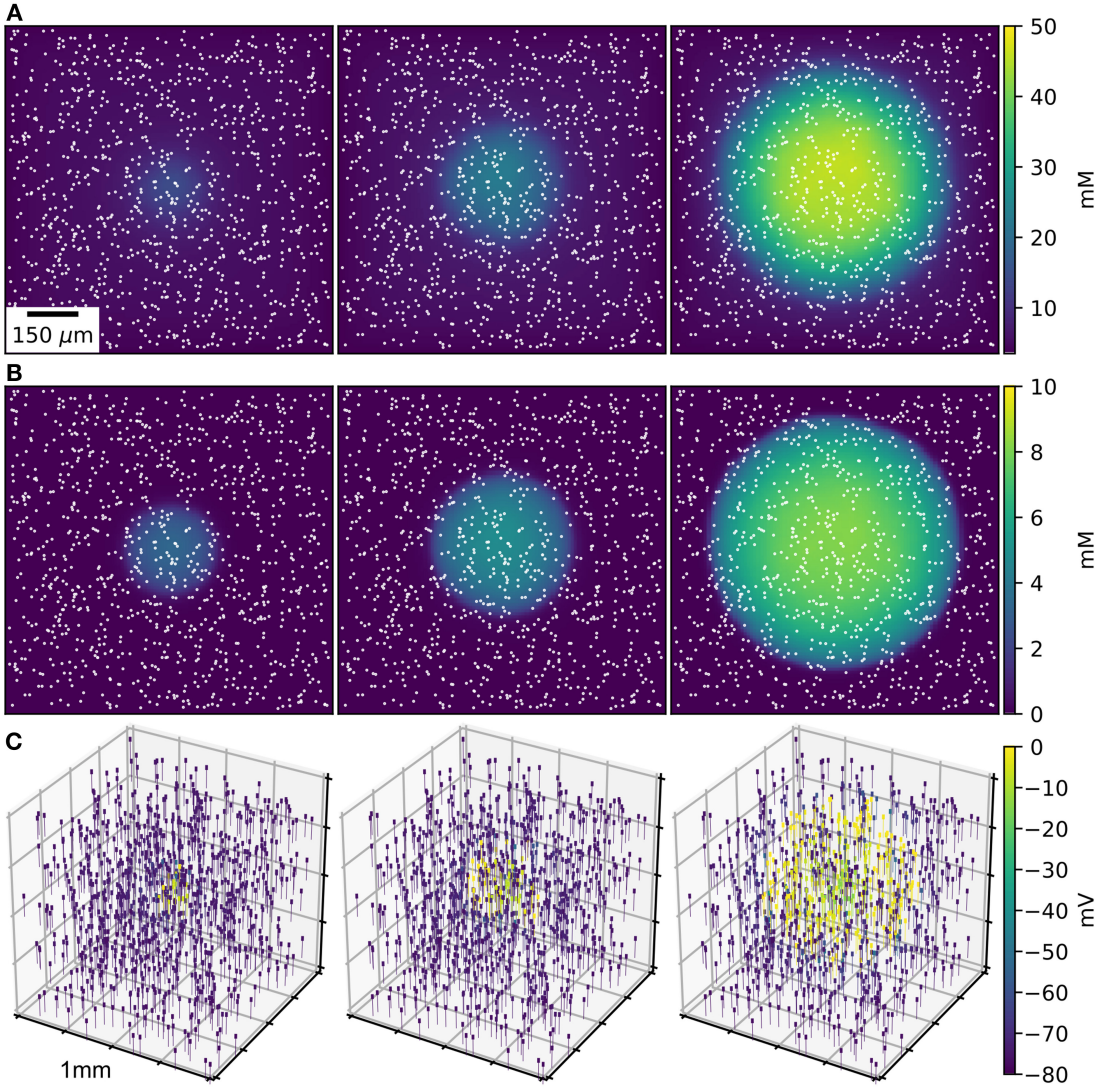
SD wave Time points at 10, 20, 30 s, with concentrations averaged over the depth of 1 mm^3^ of ECS. **(A)** Extracellular K^+^ with glial uptake and Dirichlet boundary conditions. **(B)** Glial uptake occupancy. **(C)** Membrane potential for 1,000 of the 90,000 cells [their locations are shown in **(A,B)** by white points]. Video available in Supplementary Data.

**Figure 2:**
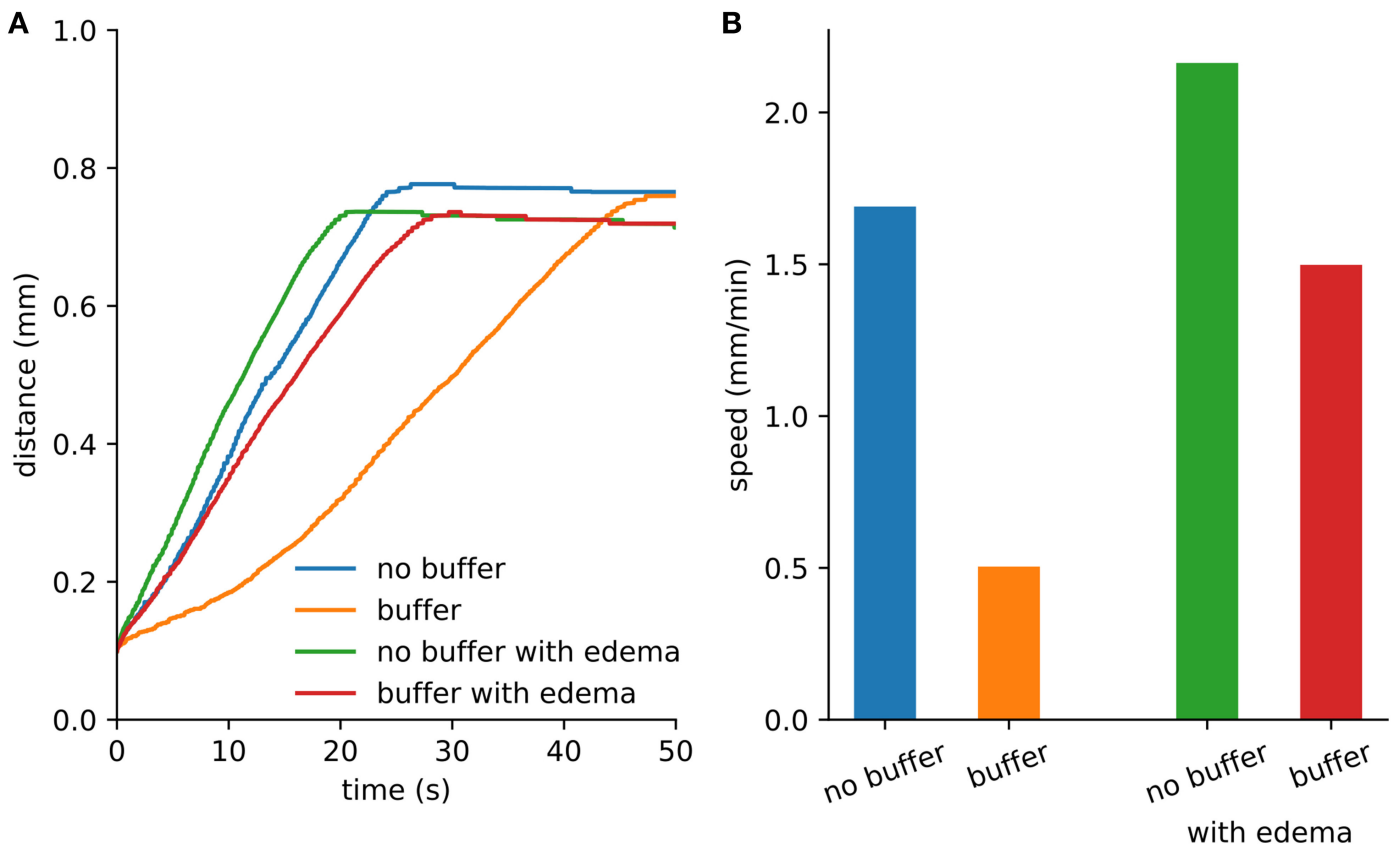
Spreading depression spread faster with edema. **(A)** Maximum distance from the center where extracellular K^+^ exceeds 15 mM. The extent was limited by the loss of K^+^ at the Dirichlet boundary. **(B)** Wave speed from the first 10 s of SD.

An initial spherical bolus of 40 mM K^+^ of radius 100 μm was placed in the center of the ECS to trigger SD. In the absence of astrocytic uptake, the SD wave front propagated at 1.69 mm/min. High astrocyte capacity of 500 mM (Bazhenov et al., 2004) immediately removed the free K^+^, preventing SD. At a far lower astrocyte density of 10 mM, SD did occur (Figure 1). SD speed was reduced by 70% compared to the no-astrocyte case (Figure 2).

#### 3.2.1. Cerebral edema

The volume-averaged macroscopic description of tissue can be characterized by free volume fraction and tortuosity. Both vary across brain regions (Nicholson and Syková, 1998), as well as during the sleep-wake cycle (Xie et al., 2013) and under pathological conditions (Hrabětová and Nicholson, 2000). A major pathological condition that decreases free volume fraction and increases tortuosity is cytotoxic edema, which is caused by cell swelling resulting in reduced ECS. In the case of ischemia (stroke), edema will be greatest at the ischemia core, the central location where metabolites have been cut-off through lack of blood flow. At the core we reduced free volume fraction to 0.07 and increased tortuosity to 1.8 (Zoremba et al., 2008). Outside of the core, there is a penumbra where cell function and ECS characteristic are less abnormal. The penumbra in turn is surrounded by normal tissue. The notion of 3 concentric volumes is a gross approximation since there is fall-off of damage as one passes from central core to normal tissue at the outside. We therefore simulated SD with cerebral edema using a linear change in the free volume fraction and tortuosity parameters from central core outward.

The characteristics of the ECS were specified with functions:


Lx, Ly, Lz = 1000, 1000, 1000
alpha0, alpha1 = 0.07, 0.2
tort0, tort1 = 1.8, 1.6
r0 = 100
  
def alpha(x, y, z) :
  return (alpha0 if x**2 + y**2 + z**2
  <  r0**2
  else min(alpha1, alpha0 +(alpha1
  -alpha0) *((x**2+y**2+z**2)**0.5-r0)/
  (Lx/2)))
  
def tort(x, y, z) :
  return (tort0 if x**2 + y**2 + z**2
  <  r0**2
  else max(tort1, tort0 - (tort0
  -tort1) *((x**2+y**2+z**2)**0.5-r0)/
  (Lx/2)))
  
ecs = rxd.Extracellular(-Lx/2.0, -Ly/2.0,
  -Lz/2.0, Lx/2.0, Ly/2.0, Lz/2.0, dx=10,
  volume_fraction=alpha, tortuosity=tort)


We repeated the SD simulation in the ischemic context. Although diffusion was slowed by the increased tortuosity, the effect was less than the speed-up obtained due to reduced volume fraction. With the reduced volume fraction, less K^+^ was required to propagate the wave (Figure 2).

This simple model demonstrates the utility and simplicity of the expanded *rxd* module. However, it only included diffusion of K^+^ and Na^+^. Other relevant species could be added to make the simulation more closely comparable to the clinical situation. Adding glutamate would produce further depolarization through synaptic receptors and could contribute to both excitotoxicity (cell damage due to excessive depolarization and calcium) and to the propagation of SD (Kager et al., 2000; Hübel et al., 2017). Demonstrating excitotoxicity would also suggest adding diffusion of calcium, which is also involved in the induction and propagation of SD. Chloride contributes to K^+^ homoeostasis via Cl-K cotransport and also regulates cell osmolarity (Hübel and Ullah, 2016).

In order to explicitly simulate uptake by astrocytes rxd.MultiCompartmentReaction would be used to define stoichiometrically-defined flux between intracellular and extracellular regions. A more sophisticated model of astrocytes would include gap junctions, allowing astrocytes to maintain a lower membrane potential facilitating K^+^ uptake. Such a model could also include spatial buffering, where K^+^ is transported via astrocytes rather than diffusion in the ECS (Gardner-Medwin, 1983). While the buffering in this simple model is neuroprotective, astrocytes also play an adverse role in SD, as gap-junction mediated calcium waves may be related to the initiation and amplification of SD, facilitating propagation over longer distances (Nedergaard et al., 1995).

These simulations focused on the wave of cell depolarization and omitted the silencing of electrical activity that follows—looking at the spreading depolarization rather than at the specifics of the spreading depression itself (Dreier, 2011). This second phase of neuronal inactivity may be related to depolarization blockade, as well as to synaptic plasticity and the accumulation of extracellular adenosine (Frenguelli and Wall, 2016; Cozzolino et al., 2018).

## 4. Implementation details

We provide a Python interface for specifying the model for ease of use and reproducibility; for performance reasons the numerical details are implemented in C and connected to Python using ctypes. This separation between interface and numerics allows the user to see a standard approach to modeling the ECS, where species and reactions are immediately apparent when examining a model. Parameters can be read directly from the Python code or obtained by querying the model through the Python console. In the future, parameters will also be accessible via a graphical user interface (GUI).

### 4.1. Model specification aids reproducibility

The concise, declaratory syntax for model specification has been slightly augmented since introduction of the original *rxd* module introduced with NEURON 7.3. However, all models implemented using a previous version of the *rxd* module will continue to work with the expanded version. Because of the vast difference in spatial scale between the intracellular and extracellular volumes, distinct modeling techniques are used to support diffusion in region rxd.Extracellular.

### 4.2. Finite-volume alternating direction implicit method

We used the Douglas-Gunn Alternating Direction Implicit method (DG-ADI) for diffusion in the ECS (Douglas and Gunn, 1964). DG-ADI divides each time step into three sub-steps (Equations A1–A3). The first deals with the diffusion operator in the x-direction, the second in the y-direction and the third in the z-direction. DG-ADI is computationally efficient with worst case runtime O(N) for *N* voxels. DG-ADI also provides an embarrassingly parallel workload. If the size of the extracellular space is *N*_*x*_×*N*_*y*_×*N*_*z*_, then there are *N*_*y*_×*N*_*z*_ independent operations for (Equation A1), *N*_*x*_×*N*_*z*_ for (Equation A2) and *N*_*x*_×*N*_*y*_ for (Equation A3). The finite volume method discretization (Equation A15) can be modified to account for heterogeneous diffusion coefficients and free volume fractions (Equation A16), while ensuring conservation of mass (**Figure 5A**). The details of the numerical scheme are given in Appendix A.

### 4.3. Just-in-time compiled reactions

Reaction-diffusion performance is further improved by using compiled reactions. Reactions are now parsed into C code which is compiled Just-In-Time (JIT). For example, the reaction given in section 3.1.3 produces the following C code;


#include  <math.h>
#include  <rxdmath.h>
void reaction(double* species_ecs,
    double* rhs)
{
    double rate;
    rate = -((species_ecs[2])*(0.0008))
      +(((0.0008)/(1.0+exp((
        -(species_ecs[0]-(15.0)))/(1.15))))
      *(species_ecs[1]))*(species_ecs[0]);
    rhs[1] = (-1)*rate;
    rhs[0] = (-1)*rate;
    rhs[2] = (1)*rate;
}


The 0 index of the species_ecs and rhs arrays corresponds to k, 1 to A and 2 to AK. The C code for the reactions are compiled into a dynamic library using the C compiler distributed with the operating system or distributed with NEURON. The compiled library is loaded and provides a function pointer that is used to numerically approximate the Jacobian. This allows function overloading, so the same method is used to process all extracellular reactions. The Jacobian for the reaction is solved using the Meschach library (Stewart and Leyk, 1994) included with the NEURON distribution. rxd.rxdmath supports all the mathematical functions in the math module. Most of these are defined in the GNU C Library math.h. Additional functions have been added in rxdmath.h.

### 4.4. Parallel implementation

**Figure 3:**
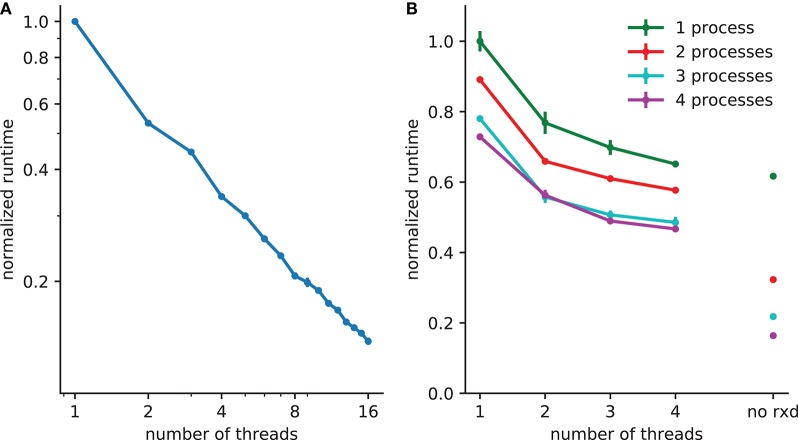
Reduction in runtime with parallelization. **(A)** 5x speedup with 8 threads with 250^3^ (15,625,000) extracellular voxels for example in section 3.1. **(B)** A spreading depression example with 1mm^3^ tissue, with 250,000 two compartment neurons and 150^3^ (3,375,000) voxels. Electrophysiology accounts for 62% of the runtime with one process (with 38% due to extracellular *rxd*), this is reduced to 22% when four processes are used, increasing the relative burden of extracellular *rxd* to 78%. Walltime minimum and standard error are shown for 5 runs of each simulation performed on a 24 core system (4 Intel Xeon L5640 processors).

Extracellular reaction-diffusion benefits from two forms of parallelization; multithreading and multiprocessor (Figure 3). Multithreading, implemented with POSIX threads, uses shared memory. The number of *rxd* threads n can be set by calling rxd.nthread(n).

A thread pool is created at the start of the simulation; the calculations required for both diffusion (DG-ADI) and reactions are distributed across the available threads in the pool. The *rxd* threads are independent of NEURON threads used for electrophysiology, which are accessed via ParallelContext.nthreads. Independent pools are used because these are independent problems: (1) electrophysiology: ParallelContext threads split computations either by cell, or by cell section (multi-split method; Hines et al., 2008) (2) diffusion and reaction: DG-ADI. Although DG-ADI is trivially parallelizable, we do not achieve optimal scaling (Figure 3A). Performance is limited by the overhead of the relatively large non-contiguous memory access required, and the need to coordinate with the NEURON time step.

The multiprocessor approach, implemented with the Message Passing Interface (MPI) is primarily intended for large neuronal network models. The network that is embedded within the ECS may in this case be purely electrophysiological or may also include intracellular *rxd*. In either case, the speed-up from using MPI is entirely due to network speed-up; each processor solves the entire ECS reaction-diffusion space independently. All cellular influx and efflux are made available to all processors. This simple approach was adopted after demonstrating that communication overhead dominated over calculation when the ECS was split across processors. Multiprocessor and multithreading can be used together, with MPI reducing the runtime for the intracellular *rxd* for electrophysiology and for networks, multiple threads reducing runtime for ECS reaction-diffusion (Figure 3B).

## 5. Verification and validation

We verified the numerical implementation by (1) comparing a simple model with its analytic solution; and (2) confirming conservation of mass, (3) comparing results with FiPy, a finite volume PDE solver (Guyer et al., 2009).

### 5.1. Comparison with analytic results

**Figure 4:**
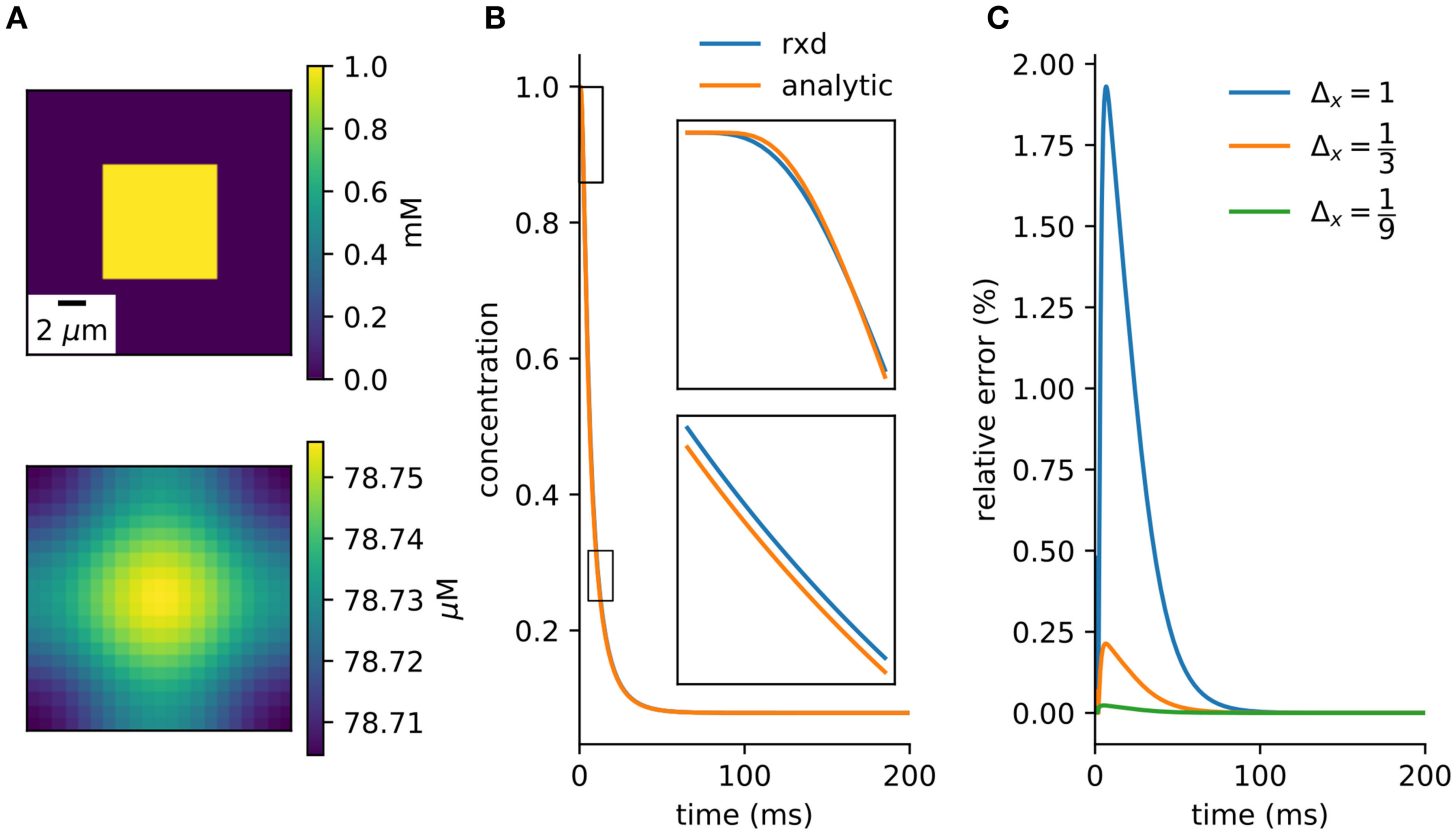
Verification against analytic solution. **(A)** Cross-section of initial conditions (top) and after 100ms (bottom). 9 μm^3^ cube diffuses in a 21 μm^3^ cube. **(B)** Concentration of center voxel compared to analytic solution (Equation A20), with insets at two 2.5 ms periods (Δ*x* = 1 μm, Δ*t* = 0.1 ms). **(C)** Relative error tends toward zero with finer spatial discretization.

A simple model with an analytic solution is an initial cube of elevated concentration diffusing in a closed boxed. It is solved by integrating the Green's function over the initial conditions and matching the Neumann boundary conditions with the method of images (Appendix B). A direct comparison to the numerical method is obtained by integrating over the central voxel and dividing by volume to obtain the average concentration at the center (Equation A20). There is close agreement between the numerical solution provided by the *rxd* module and this analytic solution (Figure 4).

### 5.2. Conservation of mass

**Figure 5:**
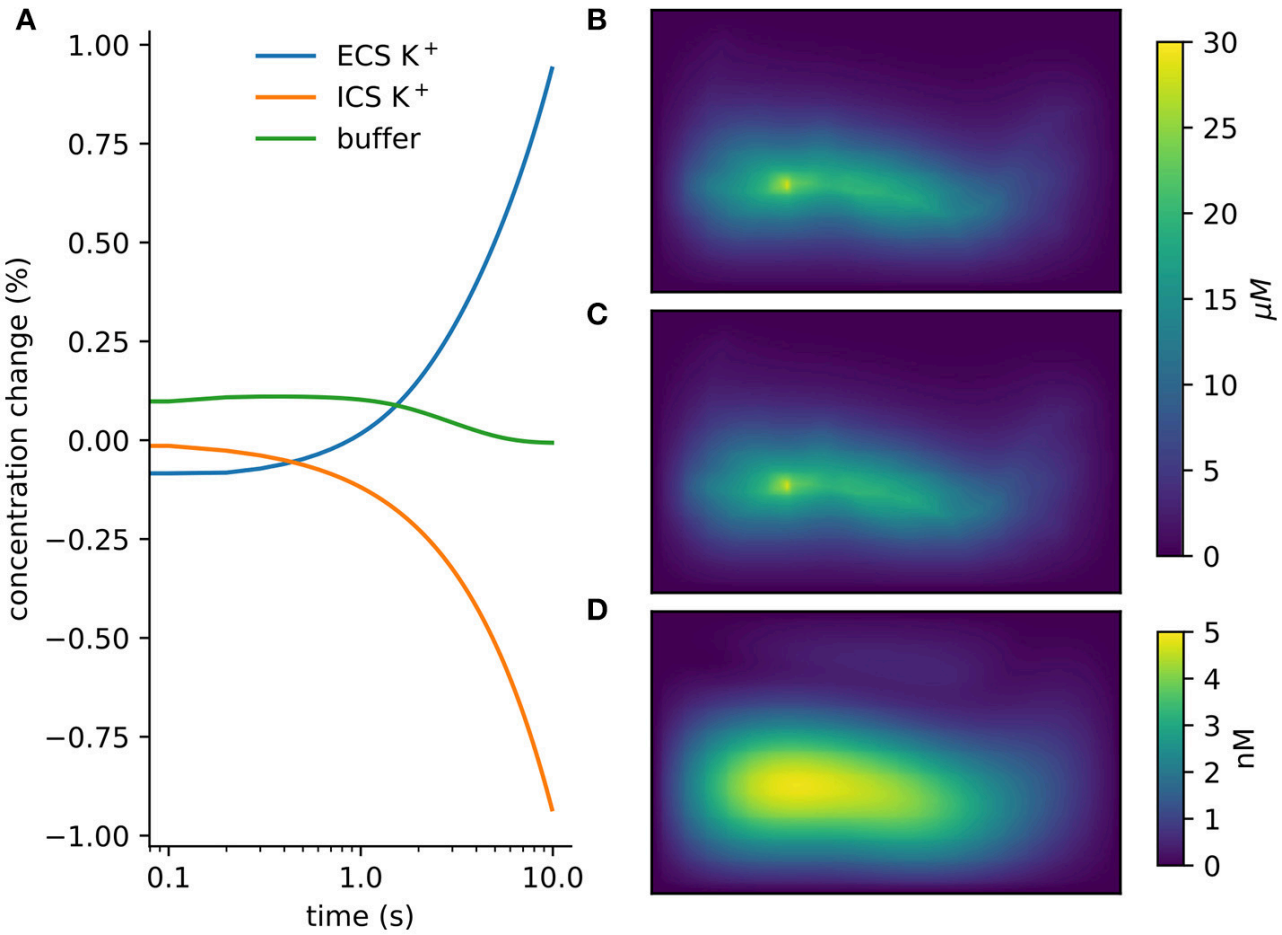
Verification and validation. **(A)** Conservation of mass for verification: < 10^−12^ change with currents from 1, 000 neurons in tissue with heterogeneous diffusion. **(B)** *rxd* simulation of 1mA/cm^2^ constant K^+^ flux from a traced rat hippocampal CA1 pyramidal neuron and Dirichlet boundary conditions, concentrations at 1 s (averaged over depth). **(C)** Comparable solution using FiPy with identical current fluxes. **(D)** Difference between *rxd* and FiPy concentrations results (note scale in nM).

When using Neumann (zero flux) boundary conditions the finite volume method will conserve mass. This provides a basic numerical and algorithmic verification that can be applied even to complex models. The example of section 3.2.1 can be modified so *rxd* manages both intracellular and extracellular concentrations. Multiplying the extracellular concentration by the volume fraction and the voxel volume and the intracellular concentration by the segment volume gives the total amount of K^+^. The change in total amount of K^+^ (Figure 5A) was on the order of floating point accuracy (~ 10^−12^).

### 5.3. FiPy comparison

We modeled a morphologically detailed reconstruction of a rat hippocampal CA1 pyramidal neuron obtained from NeuroMorpho.Org NMO_00227 (Ishizuka et al., 1995; Ascoli et al., 2007), with constant outgoing ion flux corresponding to a current density of 1mA/cm^2^ of K^+^ (Figure 5B).

In *rxd* objects provide point sources, and occupied space is represented by the free volume fraction and tortuosity. We exported the point sources from the NEURON simulation and used them with the FiPy solver (Figure 5C). Differences were ≤ 5 nM, with largest differences at the sites of efflux (Figure 5D). The sum of absolute differences was 0.03% of the total concentration at *t* = 1s.

## 6. Discussion

The original *rxd* package expanded multiscale modeling in NEURON from the electrophysiological scales of neurites, cells and networks into chemophysiological scales of spines, subcellular organelles, interactomics, metabolomics, proteomics. This further development of the module into the domain of extracellular space considerably extends the scope of chemophysiology into the vast distances of interneuronal space. Computational performance for this large-scale problem is improved by the use of multi-threading parallelization of DG-ADI algorithm for diffusion, multiprocessor parallelization for electrophysiology, and JIT compilation of reactions. The ECS module implementation was verified against an analytic solution, a test of conservation, and by comparison to an established simulator.

The extension to whole-organ simulation in the brain is particularly important for the development of multiscale modeling for clinical applications (Hunt et al., 2018; Mulugeta et al., 2018). In the past, large neural simulations have typically been neuronal networks which focus exclusively on the electrical activity of neurons and their mutual influence via chemical and electrical synapses. Such neuronal network simulations have effectively operated in a vacuum, omitting the effects of nonsynaptic neuromodulators, neuromodulatory gases, ions, glia, metabolites, etc. These physiological agents also play pathophysiological roles, for example the excessive ion concentrations seen in spreading depression, and the lack of metabolites that causes tissue damage from ischemia and stroke. Pharmacological agents used in treatments are also broadcast diffusively, as are agents and effects associated with microglia. Mechanical factors from brain trauma and current in electrical stimulation follow their respective tissue impedance boundaries. Many pathological disorders, particularly stroke and traumatic brain injury, involve large volumes of tissue. For this reason, the initial development of our new extension has focused on coarse spatial discretization in order to accommodate large distances, permitting representative neuronal networks to be seeded in a mosaic of locations within the volume.

### 6.1. Large volume averaged approach

Electrophysiological models in NEURON can specify currents either in absolute terms or as current densities. In the latter case, membrane surface area must be used to calculate the current. The ECS *rxd* module identifies ion flux from currents, which are then placed in the corresponding voxel of the ECS simulation. Macroscopic measure of ion diffusion in bulk tissue observed experimentally with ion selective sensors, biosensors, and fast-scan cyclic voltammetry can be used to constrain parameters (Budygin et al., 2000; Dale et al., 2005; Nicholson and Hrabětová, 2017).

Currently, we support two boundary conditions: Neumann boundary conditions (constant boundary flux) and Dirichlet conditions (constant boundary concentration). Neumann boundary conditions are appropriate for *in-vivo* models where we are simulating a piece of brain in continuity with other similar pieces of brain. In this case, any substance that leaves the simulated space would be replaced by substance from neighboring regions. Conversely Dirichlet conditions (constant boundary) are appropriate if the region modeled is not representative, as occurs under pathological conditions such as the core area of a stroke. In this case perturbations in extracellular concentrations are expected to be restored sufficiently far from their source. In both cases, clearance can still be modeled using NEURON models or extracellular reactions to represent transport through the blood-brain-barrier. If the ECS is made large enough relative to simulation duration, the choice of boundary conditions will not have a significant effect on results.

### 6.2. Multiple uses of extracellular reaction-diffusion simulation

There are many forms of extracellular extra-synaptic signaling between cells. Here we have illustrated the utility of the module with a simple model for spreading depression, where the “signal” is a change in ion concentration. The extracellular *rxd* module has a wide range of potential applications tracking the variety of substances of both physiological and pathological relevance. For example, neurotoxic substances such as free radicals diffuse away from areas of damaged tissue; amyloid-β oligomers may diffuse away from specific cells creating misfolding of protein in remote cells (Waters, 2010). Both synaptic spillover and nonsynaptic release provide diffusing of neurotransmitters (e.g., glutamate and excitotoxicity), and of neuromodulators: dopamine, acetylcholine, norepinephrine, adenosine, etc. For example, dopamine (DA) in striatum is released by axonal projections from midbrain, and diffuses in a local region before reuptake by DA transporter (Sulzer et al., 2016). Models of striatal activity in physiological (Humphries et al., 2010) or pathological conditions (Migliore et al., 2008; Blackwell et al., 2018) would benefit by including this extracellular dopamine spread. Simulating extracellular dopamine would follow the same procedure described in section 3.1; specifying the region with tortuosity and porosity, the species with its diffusion coefficient and boundary conditions and the reactions that remove it from the ECS including the kinetics of DA transporters.

### 6.3. Future development

The ECS simulation developed here will provide the broadest spatial scale for future multiscale models that will add additional methods at smaller scales. These multiple methods will interconnect so as to be used together in single multiscale simulations that coordinate a broad range of spatial and temporal scales, that could not be assessed using a uniform fine discretization, or uniform algorithms throughout. At the finest scales, stochastic methods will be used to better understand the variability seen at small scale, for example in synaptic clefts. Additional simulation method currently being addressed include techniques for understanding bulk tissue current flow to simulate deep and transcranial current stimulation. Whether induced externally or produced by local field potentials (Lindén et al., 2014), bulk electric field effects will not only depolarize or hyperpolarize cells, but will also affect diffusion of ions and other charged species i.e., the phenomenon of electrodiffusion. Not only are ions affected by the field, ions also produce a field that will affect other ions, potentially producing fields of order hundreds of microvolts over 1mm of tissue (Halnes et al., 2016; Solbrå et al., 2018). Such gradients are likely to have an even greater influence in SD, where there is a large redistribution of ions.

There are a number of other important organ-level processes that are particularly important for brain pathology. These include blood flow which is of importance for understanding stroke, and mechanical properties of importance for understanding traumatic brain injury. Additional processes that are unique to the brain would include CSF production, flow and reuptake; and status of the blood-brain barrier. More controversial is the role of advection—fluid flow. The brain lacks a lymphatic system for waste clearance, and the small spaces between cells, ~40 nm, are too small to support advection (Jin et al., 2016; Holter et al., 2017). It has been hypothesized to instead use a *glymphatic system* that establishes fluid flow via glial astrocytic aquaporin-4 channels, driven by pulsations from respiration and heartbeat. Fluid would flow via astrocytes oriented to provide the pathways that cannot be supported by the interstitium (Iliff et al., 2012).

All of these processes are currently the subject of multiscale modeling at varying degrees of sophistication (Anderson and Vadigepalli, 2016; Linninger et al., 2016; Calvetti et al., 2018; Durka et al., 2018; Zhao et al., 2018). Although it would not be practical to incorporate these many types of simulation within NEURON, there will be possibilities for cross-simulator communication providing complex multiphysics simulations in the future (Djurfeldt et al., 2010). In the meantime, some aspects of this complexity can be readily incorporated without considering the details: for example, brain vascularization can be modeled as a “metabolite field” that would take account of the greater availability of oxygen and glucose at locations within, and reduced availability in the *watershed areas* that lie between, the major artery distribution trees.

The term *mosaic modeling* may be used to describe these complex multiscale, multiphysics, multialgorithmic, multidimensional simulations—the mosaic involves pieces of a cell or of a brain simulated with different dimensionality, different algorithms, and different discretizations. An example at the cellular level are spines, which are best handled stochastically and in three dimensions, while the rest of the cell is handled deterministically and as a one dimensional branched tree structure (Lin et al., 2017a,b). Similarly, in the ECS, small spaces such as synapses require a microscopic approach that is not practical for bulk tissue modeling. In the future, these pieces of the mosaic will be adapted from approaches currently used by other simulators. For example, one approach at small scales is to track individual particles, done by Smoldyn (Andrews, 2012) and MCell (Stiles and Bartol, 2001; Franks et al., 2002). Another small-volume technique uses averaged volumetrics as done by the ENOS platform, which has also been used for high resolution models of glutamatergic synapses and their interaction with glia (Bouteiller et al., 2008). Other platforms that support intracellular diffusion will also be mined for additional techniques, including STEPS (Wils and De Schutter, 2009), NeuroRD (Brandi et al., 2011), MOOSE (Ray and Bhalla, 2008).

## Author contributions

WL, MH, RM, and AN expanded the rxd module. WL, RM, and AN created the examples and wrote the paper.

### Conflict of interest statement

The authors declare that the research was conducted in the absence of any commercial or financial relationships that could be construed as a potential conflict of interest.

## A. Heterogeneous tortuosities and volume fractions

Solving the diffusion equation in 3D with DG-ADI method, involves splitting the problem into 3 linear equations for each time-step;

(A1) $$\begin{array}{l} 1-\frac{r_{x}}{2}\nabla_{x}^{2}\phi^{\left(j+\frac{1}{3}\right)}=\left(\frac{r_{x}}{2}\nabla_{x}^{2}+r_{y}\nabla_{y}^{2}+r_{z}\nabla_{z}^{2}\right)\phi^{\left(j\right)} \end{array}$$

(A2) $$\begin{array}{l} 1-\frac{r_{y}}{2}\nabla_{y}^{2}\phi^{\left(j+\frac{2}{3}\right)}=-\frac{r_{y}}{2}\nabla_{y}^{2}\phi^{\left(j+\frac{1}{3}\right)} \end{array}$$

(A3) $$\begin{array}{l} 1-\frac{r_{z}}{2}\nabla_{z}^{2}\phi^{\left(j+1\right)}=-\frac{r_{z}}{2}\nabla_{z}^{2}\phi^{\left(j+\frac{2}{3}\right)} \end{array}$$

Where $r_{x}=\frac{D\Delta_{t}}{\Delta_{x}^{2}}$, $r_{y}=\frac{D\Delta_{t}}{\Delta_{y}^{2}}$ and $r_{z}=\frac{D\Delta_{t}}{\Delta_{z}^{2}}$. Δ_*x*_, Δ_*y*_, Δ_*z*_ and Δ_*t*_ are the spatial and temporal discretization step sizes and *D* is the diffusion coefficient. The variables *ϕ*^(*j*)^ and *ϕ*^(*j*+1)^ are the concentrations at the *j* and *j* + 1 time-step and $\phi^{\left(j+\frac{1}{3}\right)}$ $\phi^{\left(j+\frac{2}{3}\right)}$ are intermediate solutions that do not correspond to a concentration at a given time. Each equation involves the Laplacian (∇^2^) for a different dimension ($\nabla_{x}^{2}$, $\nabla_{y}^{2}$, or $\nabla_{z}^{2}$). So to adapt DG-ADI method for inhomogeneous tortuosities or volume fractions, it is sufficient to consider how to modify the 1D diffusion operator.

### A.1. tortuosity

The diffusion equation (in one dimension) with an inhomogeneous tortuosity (λ) is;

(A4) $$\begin{array}{l} \frac{\partial \phi \left(t,x\right)}{\partial t}=\nabla \cdot \frac{D}{\lambda {\left(x\right)}^{2}}\nabla \phi \left(t,x\right) \end{array}$$

Here we use the finite-volume method with *N* voxels with the tortuosities defined at the boundaries $\lambda_{i}=\lambda \left(x_{i-\frac{1}{2}}\right)$ and average concentrations at the centers ϕ_*i*_(*t*) = ϕ(*t, x*_*i*_) for *i* = 0, …*N* − 1. The flux at the left and right of the *i*^th^ voxel are;

(A5) $$\begin{array}{l} F_{i-\frac{1}{2}}=\frac{D}{\lambda_{i}^{2}}\frac{\phi_{i}\left(t\right)-\phi_{i-1}\left(t\right)}{\Delta_{x}} \end{array}$$

(A6) $$\begin{array}{l} F_{i+\frac{1}{2}}=\frac{D}{\lambda_{i+1}^{2}}\frac{\phi_{i+1}\left(t\right)-\phi_{i}\left(t\right)}{\Delta_{x}} \end{array}$$

This gives the semi-discretized form of the diffusion equation;

(A7) $$\begin{array}{l} \frac{d\phi_{i}\left(t\right)}{dt}=\frac{F_{i+\frac{1}{2}}-F_{i-\frac{1}{2}}}{\Delta_{x}} \end{array}$$

(A8) $$\begin{array}{l} =\frac{D}{\Delta_{x}^{2}}\left(\frac{\phi_{i+1}\left(t\right)}{\lambda_{i+1}^{2}}-\left(\frac{1}{\lambda_{i+1}^{2}}+\frac{1}{\lambda_{i}^{2}}\right)\phi_{i}\left(t\right)+\frac{\phi_{i-1}\left(t\right)}{\lambda_{i}^{2}}\right) \end{array}$$

Neumann boundary conditions with zero flux are obtained by setting $F_{-\frac{1}{2}}=F_{N-\frac{1}{2}}=0$. This discretization of the diffusion operator can then be applied to the 3D diffusion problem using DG-ADI method.

### A.2. volume fraction

A similar approach is used for inhomogeneous volume fractions, but it is important to distinguish between the total concentration (*C*_*T*_) and the relative concentration (*C*_*R*_). *C*_*T*_ is the amount divided by the volume of the voxel, *C*_*R*_ is the amount divided by the free volume of the voxel. These quantities are related by α, the volume fraction *C*_*T*_ = α*C*_*R*_. The concentration used in extracellular *rxd* are relative concentrations, as this is more biological relevant. Subsequently currents between cells and the ECS are scaled by the volume fraction.

Let both the volume fractions and the concentrations be defined at the center of the voxels α_*i*_ = α(*x*_*i*_). Then the relative concentration at the boundary, by linear interpolation is;

(A9) $$\begin{array}{l} \phi \left(t,x_{i+\frac{1}{2}}\right)=\frac{\alpha_{i+1}\phi \left(t,x_{i+1}\right)+\alpha_{i}\phi \left(t,x_{i}\right)}{\alpha_{i+1}+\alpha_{i}} \end{array}$$

Then the flux of the total concentration is given by;

(A10) $$\begin{array}{l} F_{i+\frac{1}{2}}=\frac{D}{\frac{1}{2}\Delta_{x}}\alpha_{i}\left(\phi \left(t,x_{i+\frac{1}{2}}\right)-\phi \left(t,x_{i}\right)\right) \end{array}$$

(A11) $$\begin{array}{l} =\frac{D}{\frac{1}{2}\Delta_{x}}\frac{\alpha_{i}\alpha_{i+1}}{\alpha_{i}+\alpha_{i+1}}\left(\phi \left(t,x_{i+1}\right)-\phi \left(t,x_{i}\right)\right) \end{array}$$

The fluxes are then divided by the relevant volume fraction for the semi-discretized form of the diffusion equation, i.e., for voxel *i*;

(A12) $$\begin{array}{l} \frac{r_{x}}{2}\nabla_{x}^{2}=\frac{1}{\alpha_{i}\Delta_{x}}\left(F_{i+\frac{1}{2}}-F_{i-\frac{1}{2}}\right) \end{array}$$

Note that if the volume fractions are uniform then (Equation A9, A11, A12) are;

(A13) $$\begin{array}{l} \phi \left(t,x_{i+\frac{1}{2}}\right)=\frac{\phi \left(t,x_{i+1}\right)+\phi \left(t,x_{i}\right)}{2} \end{array}$$

(A14) $$\begin{array}{l} F_{i+\frac{1}{2}}=\alpha_{i}\frac{D}{\Delta_{x}}\left(\phi \left(t,x_{i+1}\right)-\phi \left(t,x_{i}\right)\right) \end{array}$$

(A15) $$\begin{array}{l} \frac{r_{x}}{2}\nabla_{x}^{2}=\frac{1}{\Delta_{x}}\left(F_{i+\frac{1}{2}}-F_{i-\frac{1}{2}}\right) \end{array}$$

Which is the standard finite-volume approximation.

### A.3. tortuosity and volume fraction

It is straightforward to adapt the above formula for when both tortuosity and volume fraction vary, the flux term (Equation A11) is;

(A16) $$\begin{array}{l} F_{i+\frac{1}{2}}=\frac{D}{\frac{1}{2}\lambda_{i}^{2}\Delta_{x}}\frac{\alpha_{i}\alpha_{i+1}}{\alpha_{i}+\alpha_{i+1}}\left(\phi \left(t,x_{i+1}\right)-\phi \left(t,x_{i}\right)\right) \end{array}$$

## B. Analytic solution for validation

The Green's function for a source at location **x**′ = (*x*′, *y*′, *z*′) and time *t*′ is;

(A17) $$\begin{array}{l} g\left(\text{x},t,{\text{x}}^{\prime },t^{\prime }\right)=\frac{1}{{\left(4\pi D\left(t-t^{\prime }\right)\right)}^{\frac{3}{2}}}\\ \;\exp\left(-\frac{{\left(x-x^{\prime }\right)}^{2}+{\left(y-y^{\prime }\right)}^{2}+{\left(z-z^{\prime }\right)}^{2}}{4D\left(t-t^{\prime }\right)}\right) \end{array}$$

Given an initial unit concentration in a cube of size *l*^3^ at the origin, the concentrations for an unbounded space are found by integrating the Green's function.

(A18) $$\begin{array}{l} \phi_{u}\left(x,y,z,t\right)=\frac{1}{8}\left[\mathrm{erf}\left(\frac{l-2x}{\sqrt{16Dt}}\right)+\mathrm{erf}\left(\frac{l+2x}{\sqrt{16Dt}}\right)\right]\\ \left[\mathrm{erf}\left(\frac{l-2y}{\sqrt{16Dt}}\right)+\mathrm{erf}\left(\frac{l+2y}{\sqrt{16Dt}}\right)\right]\\ \left[\mathrm{erf}\left(\frac{l-2z}{\sqrt{16Dt}}\right)+\mathrm{erf}\left(\frac{l+2z}{\sqrt{16Dt}}\right)\right] \end{array}$$

For diffusion within a finite cube of volume *L*^3^ with zero flux boundary conditions, the solution is obtain by the method of images;

(A19) $$\begin{array}{l} \phi \left(x,y,z,t\right)=\overset{\infty }{\underset{i=-\infty }{\sum }}\overset{\infty }{\underset{j=-\infty }{\sum }}\overset{\infty }{\underset{k=-\infty }{\sum }}\phi_{u}\left(x+iL,y+jL,z+kL,t\right) \end{array}$$

So the average concentration for a voxel of size $\Delta_{x}^{3}$ at the center is;

(A20) $$\begin{array}{l} \phi_{0}(t)=\frac{1}{8\Delta_{x}^{3}}\sum_{i=-\infty }^{\infty }\sum_{j=-\infty }^{\infty }\sum_{k=-\infty }^{\infty }\prod_{m\in \{i,j,k\}}\\ \;\sqrt{\frac{16Dt}{\pi }}[\exp\left(-\frac{{\left(l+2mL+\Delta_{x}\right)}^{2}}{16Dt}\right)\\ \;-\exp-\frac{{\left(l+2mL-\Delta_{x}\right)}^{2}}{16Dt}]\\ \;+(l+2mL+\Delta_{x})\text{erf}\left(\frac{l+2mL+\Delta_{x}}{\sqrt{16Dt}}\right)\\ \;+(l+2mL-\Delta_{x})\text{erf}\left(\frac{l+2mL-\Delta_{x}}{\sqrt{16Dt}}\right) \end{array}$$

The terms of the sum decay with order *e*^−*m*^2^^ so few are needed.
